# Correction: Mano et al. Fluidity of Poly (ε-Caprolactone)-Based Material Induces Epithelial-to-Mesenchymal Transition. *Int. J. Mol. Sci.* 2020, *21*, 1757

**DOI:** 10.3390/ijms27010536

**Published:** 2026-01-05

**Authors:** Sharmy Saimon Mano, Koichiro Uto, Mitsuhiro Ebara

**Affiliations:** 1 International Center for Materials Nanoarchitectonics (WPI-MANA), National Institute for Materials Science (NIMS), 1-1 Namiki, Tsukuba 305-0044, Ibaraki, Japan; 2 International Center for Young Scientist (ICYS), National Institute for Materials Science (NIMS), 1-1 Namiki, Tsukuba 305-0044, Ibaraki, Japan; 3 Graduate School of Pure and Applied Sciences, University of Tsukuba, 1-1-1 Tennodai, Tsukuba 305-8577, Ibaraki, Japan; 4 Graduate School of Tokyo University of Science, 6-3-1 Niijuku, Katsushika-City 125-8585, Tokyo, Japan

In the original publication [1], there was a mistake in Figure 1a, in which an incorrect image was inadvertently included due to a misplacement during layout processing (80 *w*/*v*%). We would like to emphasize that the image itself remained unaltered and the error was limited solely to its placement within the manuscript. The corrected 80 *w*/*v*% appears below, accurately depicting the intended data visualization. This amendment does not affect the experimental findings, data analysis, or conclusions drawn in the study. The authors state that the scientific conclusions are unaffected. This correction was approved by the Academic Editor. The original publication has also been updated.

**Figure 1:**
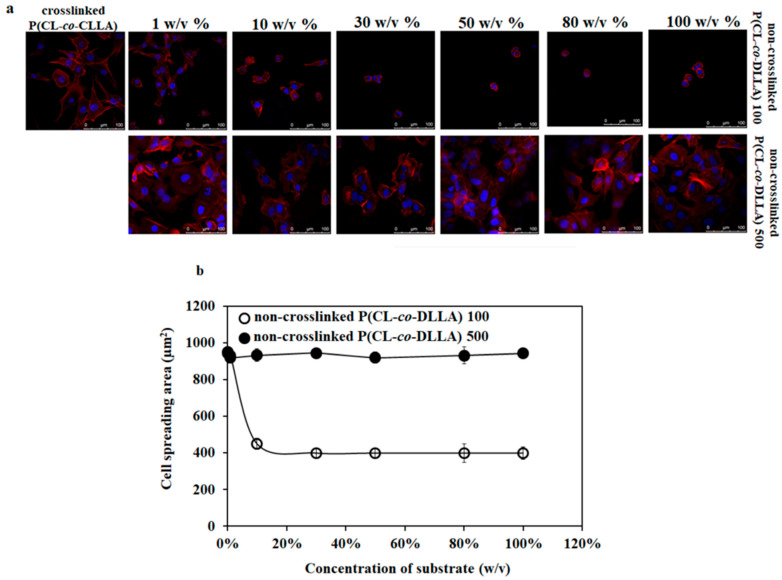
(**a**) Confocal microscopic images of cell spreading behavior of MCF-7 cells on crosslinked and non-crosslinked P(CL-*co*-DLLA) 100 or 500 substrates of different concentrations (1–100 *w*/*v*%). The spreading behavior of MCF-7 cells decreases with an increased concentration of non-crosslinked P(CL-*co*-DLLA) 100 substrates while the morphology of MCF-7 cells remains unchanged on any concentration of non-crosslinked P(CL-*co*-DLLA) 500 substrates. (**b**) Graphical representation of the area of cells on crosslinked and non-crosslinked P(CL-*co*-DLLA) substrates. The cell spreading area decreases when the concentration of P(CL-*co*-DLLA) 100 substrates (white circles) increases and the cells remain in the elongated morphology in any concentration of P(CL-*co*-DLLA) 500 substrates (black circles). Fifty cells were counted for each sample and are represented with ± SD.
